# Firearm availability and police shootings of citizens: a city level analysis of fatal and injurious shootings in California and Florida

**DOI:** 10.1186/s40621-023-00466-1

**Published:** 2023-10-20

**Authors:** John A. Shjarback, Daniel C. Semenza, Richard Stansfield

**Affiliations:** 1 Department of Law and Justice Studies, Rowan University, 201 Mullica Hill Road, Glassboro, NJ 08028 USA; 2 Department of Sociology, Anthropology, and Criminal Justice, Rutgers University-Camden, Camden, NJ USA; 3 Department of Urban-Global Public Health, Rutgers University, Piscataway, NJ USA; 4 New Jersey Gun Violence Research Center, Piscataway, NJ USA

**Keywords:** Police shootings, Firearms, Firearm availability, Fatalities, Injuries

## Abstract

**Background:**

A growing body of research has found a link between firearm availability and police shootings of citizens across place. The problem, however, is that the previous studies on the topic tend to suffer from several limitations: a near exclusive focus on citizen fatalities, units of analysis at the state or county levels, and a variety of proxy measures tapping into community-level firearm access. The current study set out to address these issues by examining the relationship between different forms of firearm availability and both fatal and nonfatal injurious police shootings of citizens at the city level.

**Methods:**

More specifically, it merged *The Trace*’s “Missing Pieces” measures of guns reported lost and stolen to police as well as licensed firearms dealers across jurisdictions from the Bureau of Alcohol, Tobacco, Firearms, and Explosives as proxies for firearm availability with data on police shootings of citizens in California and Florida from California’s URSUS system and the *Tampa Bay Times*’ “Why Cops Shoot” database, respectively. Negative binomial regression analyses were performed on a sample of 253 cities across the two states and a sub-sample of cities with licensed firearms dealers.

**Results:**

Findings uncovered a small positive association between rates of federally licensed guns stores and the number citizens shot by police as well as police shooting incidents while controlling for several community-level measures (e.g., concentrated disadvantage, gun homicide rates). Rates of guns lost or reported stolen were generally not significantly associated with the outcome measures in the multivariate models.

**Conclusions:**

Firearm availability is a significant correlate of police shootings. Pooled counts of both citizens shot by police and police shooting incidents are heightened in jurisdictions with higher rates of licensed gun dealers, which may be due to the fact that all firearms sold in the USA first make their way to the public through these mechanisms. Such licensed gun dealers must be appropriately monitored and audited to reduce illicit behavior and prevent firearms from making their way into secondary markets. Addressing access to firearms can be meaningful for a host of gun-related morbidity and mortality outcomes, including police shootings of citizens.

**Supplementary Information:**

The online version contains supplementary material available at 10.1186/s40621-023-00466-1.

## Background

Scholarship on police shootings has expanded significantly in the last few years due, in large part, to the advancement of data collections tracking citizen deaths at the hands of officers (e.g., *The Washington Post*’s “Fatal Force”, Mapping Police Violence, “The Counted” by *The Guardian*) (Williams et al. 2019). Police officers in the United States (US) fatally shoot approximately 1000 people each year or roughly 2–3 every day (Conner et al. 2019). Researchers have found that the presence of a weapon, specifically a firearm, is one of the strongest, most consistent predictors of police shootings at the individual and situational levels (Alpert and Dunham 2004; Phillips and Kim 2021; Wheeler et al. 2017). More broadly, firearm access and related legislation have also been associated with fatal police shootings. Rates of police shooting deaths of citizens are higher in states that have greater household gun ownership (Hemenway et al. 2019; Nagin 2020).^1^ Lower rates of fatal police shootings, conversely, occur in states with laws aimed at strengthening background checks (e.g., permit-to-purchase or PTP laws), promoting safe storage, and reducing gun trafficking (Kivisto et al. 2017; Crifasi et al. 2023). Further, more relaxed laws regulating guns in states, such as permitless concealed carry for citizens, are associated with higher rates of both fatal and injurious police shootings (Crifasi et al. 2023; Doucette et al. 2022). Taken together, a growing body of studies have established a link between various aspects of firearm access and the likelihood of police shootings of citizens.

Despite extant work, however, there remain at least three significant limitations to research on the issue of firearm access and police shootings. First, the vast majority of studies on the topic focus exclusively on fatal police shootings of citizens (Shane et al. 2017; Sheppard et al. 2022). Fatalities represent a subset of all police shootings and, thus, deadly force incidents which are defined as “any physical force that is capable of or likely to kill; it does not always kill” (Fyfe 1978). The number of people who survive their gunshot wounds is likely hundreds if not close to 1000 more each year beyond those that are fatally shot by US police (Nix and Shjarback 2021; Shjarback 2019; Ward 2023).^2^ As such, an emphasis solely on fatal shootings by the police minimizes the much broader impact that police shootings have on the public and throughout local communities.

Second, prior studies—particularly those that utilize states or counties as the unit of analyses—are likely to be hampered by aggregation bias (Ratcliffe and McCullagh 1999), which suggests there is more opportunity to conceal variation across geographic spaces that is less visible at larger levels of aggregation. State and even county-level units of analysis likely have a large mix of urban, suburban, and rural areas with vastly different population demographics within those spatial boundaries. Although data availability and limitations remain, an examination of firearm access and police shootings are ultimately better suited to include smaller units of analysis such as cities—particularly if data do not permit researchers from examining more spatially specific levels of aggregation (e.g., census tracts, block groups).

Third, although studies on the issue of firearm access and police shootings have relied on a wide variety of measures of firearm access and availability (Hemenway et al. 2019), no study to our knowledge has leveraged separate measures to differentiate between legal access to guns (via federally licensed firearm dealers or FFLs) versus access to illicit weapons through secondary markets that include stolen or lost firearms. This is a critical distinction that cannot be captured using standard proxy measures of firearm access such as the percentage of suicides committed with a gun (Nagin 2020) or survey-based measures of firearm ownership in the home (Azrael et al. 2004). Similarly, measures of firearm legislation as proxies for legal stringency cannot fully account for different types of access to guns. Examining and better understanding the method(s) of firearm access as it relates to police shootings of citizens is critical from a public health perspective.

Research suggests that access to firearms via firearm dealers versus illicit markets can differentially influence violent outcomes. For instance, a study of homicides in 226 US cities found that illicit access to firearms is more pertinent to general homicide rates than legal access via firearm dealers (Semenza et al. 2021). This finding corresponds to a broader body of literature that suggests individuals embedded in high-risk social networks commit gun violence most often using firearms acquired through theft, diversion from gun stores, or informal trade and gifting (Braga et al. 2021; Cook et al. 2007; Papachristos et al. 2013). As such, easier access to illicit guns is likely to increase the risk of criminal homicide in a given area. However, other studies have shown that the prevalence and local density of FFLs correspond with higher rates of various types of killings including intimate partner homicides (Semenza et al. 2021, 2022a; Johnson and Robinson 2021; Stansfield and Semenza 2019; Stansfield et al. 2021; Wiebe et al. 2009).

Although firearm availability in general corresponds to more fatal police shootings of citizens (Hemenway et al. 2019; Nagin 2020), the context of access to guns in a given city may be especially important depending on the likelihood of an officer encountering someone with a firearm (and subsequently either non-compliant or threatening with that said weapon) as well as an officer’s perception of injury risk during an altercation (Doucette et al. 2022). This differentiation between legal versus illicit firearm access is purely exploratory, especially considering the fact that little-to-no quantitative research has examined officers’ perceptions of firearm risk or danger more broadly. Further, the presence of different types of dealers (e.g., pawnbrokers versus “Big Box” stores like Bass Pro Shops or Walmart) may differentially signal firearm access and availability to police if they know citizens can easily obtain a gun from a given store nearby (Steidley et al. 2017). On the other hand, the supply of illicit firearms may be more salient for police interactions if officers either confront or are aware that people can easily access stolen weapons through secondary underground markets (Cook et al. 2007). Since people who engage in violent crime are most likely to use illegally obtained firearms (Braga et al. 2021), cities with greater supplies of illicit firearms may experience higher rates of police shootings.

The current study addresses limitations of past research by examining the relationship between both legal and illicit firearm availability as well as fatal and nonfatal injurious (i.e., struck by gunfire) police shootings of citizens at the city level. We merge data from *The Trace*’s “Missing Pieces” project, which provides measures of guns reported lost or stolen to law enforcement agencies across cities as a proxy for illicit firearm availability, with detailed information on FFLs from the Bureau of Alcohol, Tobacco, Firearms, and Explosives (ATF) and two data sources—California’s URSUS system and the *Tampa Bay Times*’ “Why Cops Shoot” database—that capture fatal and nonfatal injurious police shootings in California and Florida, respectively.

## Methods

### Data and sample

We merged data from several sources, including police shootings of citizens in Florida and California, *The Trace*’s “Missing Pieces” collection, ATF, and the US Census Bureau’s American Community Survey (ACS). Measures of both fatal and nonfatal injurious police shootings in Florida and California are publicly available through the *Tampa Bay Times*’ “Why Cops Shoot” database and the California Department of Justice’s URSUS, respectively.^3^ Using freedom of information and media requests as well as court documents, this journalist-compiled effort records those shot by police (i.e., fatal and injurious) from January 1, 2009 through the end of 2014 throughout Florida. Starting January 1, 2016, California’s URSUS collects all police firearms discharges throughout the state. In an effort to keep consistent measures with Florida, all fatal and injurious police shootings of citizens were included through the end of 2021. These data have been used in a number of prior studies (Nix and Shjarback 2021; Nazaretian et al. 2021; Premkumar et al. 2021).

The Trace’s “Missing Pieces” project combines over 800,000 records on guns reported lost, stolen, or recovered by law enforcement. The Trace, an independent nonprofit news organization, compiled these data with the assistance of several local media partners by filing public records requests with major cities around the US, covering over 1000 police agencies. These data have been used in recent prior studies (Semenza et al. 2021; Stansfield et al. 2021) to capture the number of lost or stolen guns, often connected to crime scenes, yet it is worth noting that the measures—as a whole—have not been independently validated for reliability in other peer-reviewed publications. The data, nevertheless, do reflect broader national trends in firearm thefts shown by the National Crime Information Center (NCIC) (Freskos 2017). The Trace data also capture the number of guns recovered by police agencies that were not previously reported stolen or lost. However, since these measures may be subject to the level of proactive policing efforts at the department-level or heterogeneity in reporting methods—which could, arguably, bias them more than the number of guns lost or reported stolen—they were excluded from our analysis. The Trace’s data included a total of 253 jurisdictions in Florida (*n* = 57; 22.5%) and California (*n* = 196; 77.5%)—where the unit of analysis is the city as opposed to the census tract, county, etc.

#### Measures

The primary dependent variables are pooled counts of fatal and nonfatal injurious police shootings of citizens (i.e., struck by gunfire), although they are also presented as standardized rates per 100,000 citizens from 5-year estimates from the ACS (2009–2013 for Florida; 2016–2020 for California) for descriptive and zero-order correlation purposes. Given the statistically rare nature of police shootings (Ridgeway 2020), we combined 6 years’ worth of data for each jurisdiction; 15.4% of the sample (*n* = 39) had zero shootings over the study period and, cumulatively, 75.5% averaged 1 or fewer citizens shot per year. Both the total number of citizens shot as well as the total shooting incidents where multiple people might have been shot were examined.

Our independent variables represent two proxy measures of firearm availability in their respective jurisdictions. We calculated five-year average (2010–2014) rates of guns lost or stolen per 1000 citizens using 5-year estimates from the ACS (2009–2013 for Florida; 2016–2020 for California. Data from The Trace’s “Missing Pieces” contain nearly complete stolen-gun records for Florida and California—both of which have centralized sources/collections of gun-theft data—whereas measures from all other states were obtained from individual law enforcement agencies. Given the centralized sources, this should provide more confidence among cities in these two states. Next, five-year average (2010–2014) rates of federally licensed *guns stores* per 100,000 citizens were calculated using 5-year estimates from the ACS (2009–2013 for Florida; 2016–2020 for California). Such measures have been used in previous research (Semenza et al. 2021; Stansfield et al. 2021; Steidley et al. 2017) and one study in particular (Haviland et al. 2021) identified FFL rates per population as one of the best proxies for gun ownership that performed better than the proportion of suicides with a firearm. Although there is variability in the “time to crime” between guns sold and being recovered at the scene of a crime, a recent ATF report (Bureau of Alcohol, Tobacco and Firearms (ATF) 2023) found that 35% of traced crime guns are recovered with 10 miles or less from the FFLs where these firearms were acquired and the majority of crime guns recovered in both California and Florida are sourced intrastate; see also Stansfield et al. (2023) for detailed discussion of the geographic proximity of FFLs and gun crime.

In addition to the full sample, we also conducted an analysis of a sub-sample of jurisdictions where federally licensed gun stores were located (*n* = 163). As in prior studies (Steidley et al. 2017), we only focus on the two types of dealers most directly involved in the sale of a firearm to private citizens. These ATF categorizations include federal firearms license (FFL) 1 (e.g., typical local gun shop) and FFL2 (e.g., pawnshop). A measure of Big Box Stores (e.g., Walmart, Bass Pro Shop, Cabela’s) was also separately constructed from the list of FFL1s based on a manual search of duplicate names in the ATF listings. Five-year average (2010–2014) rates of *FFL1*, *FFL2*, and *Big Box Store* guns stores per 100,000 citizens were calculated using 5-year estimates from the ACS (2009–2013 for Florida; 2016–2020 for California). All of the independent variables mirror previous studies that have used The Trace’s “Missing Pieces” and ATF data (Semenza et al. 2021; Stansfield et al. 2021).

A number of variables at the city level were included as statistical controls.^4^ We created a measure of *concentrated disadvantage*, which consisted of the percentage of the population living in poverty, the percentage of the population with female-headed households, and the median income (reverse coded) from 2010. Results from a principal component analysis found that the three measures loaded on a single construct (*λ* = 2.53; factor loadings > 0.91). They were combined into a standardized weighted factor score. Each jurisdiction’s logged *gun homicide rate* per capita from 2010 was included as a measure of local gun violence in each city as well as the *percent Black* and *percent Hispanic* of the population’s jurisdiction in 2010. Lastly, a dummy variable accounting for the *state* was provided (1 = Florida).

#### Analytic strategy

First, summary statistics were used to describe all of the measures. Next, bivariate relationships (Pearson’s *r*) were assessed between the variables of interest, particularly the per capita rates of citizens shot by police and shooting incidents. Those relationships were then tested at the multivariate level using the pooled counts. Given that the dependent variables were measured as counts that were overdispersed (i.e., variances exceeding the means), the data were estimated using a series of multivariate negative binomial regressions in Stata 13 each using the cities’ population estimates from the ACS as an offset for measures of exposure. Modeling began with the full sample followed by the sub-sample of jurisdictions with federally licensed gun stores.^5^ List-wise deletion was implemented to address missing item data on a small number of cases.

## Results

Table 1 presents the summary statistics for each of the variables. Focusing first on the dependent variables, the pooled counts are used primarily for the multivariate analyses. Citizens shot range from 0 to 170 with a mean of 6.21 (SD = 14.05); shooting incidents range from 0 to 169 with a mean of 6.00 (SD = 13.60). When these measures as standardized, the *citizens shot rate* ranged from 0 to 41.92 per 100,000 with a mean of 5.08 (SD = 5.07), whereas the *shooting incidents rate* ranged from 0 to 38.11 with a mean of 4.93 (SD = 4.81). Eight out of the top ten jurisdictions with the highest rates of citizens shot were in Florida. Moving onto the independent variables, the mean *guns lost or stolen rate* was 0.39 (SD = 0.33), and it ranged from 0.04 to 2.03. The top ten highest rates of guns lost or stolen were evenly split with five jurisdictions in Florida and five in California. Gun store rates ranged from 0 to 89.47 with a mean of 6.37 (SD = 11.21). All of the top ten jurisdictions with the highest rate of gun stores per capita were located in Florida. Regarding the specific types of FFL rates for the sub-sample analyses of jurisdictions with gun stores, the mean FFL1 rate was 8.61 (SD = 10.44), followed by the FFL2 rate (mean = 1.38; SD = 3.37) and Big Box Store rate (mean = 1.00; SD = 1.28). In terms of the statistical controls, concentrated disadvantage was a weighted factor score with a mean of 0 (SD = 1), the logged gun homicide rate had a mean of 1.08 (SD = 0.91), the percent Black of the population’s jurisdiction ranged from 0 to 76.54 (mean = 8.87; SD = 11.83), while the percent Hispanic ranged from 3.02 to 98.28 (mean = 33.28; SD = 22.89).

**Table 1:** Summary statistics

Variables	Mean (SD)	Range
*Dependent variables*		
*Pooled counts (multivariate analyses)*		
Citizens shot	6.21 (14.05)	0–170
Shooting incidents	6.00 (13.60)	0–169
*Rates per 100,000 (descriptive)*		
Citizens shot rate	5.08 (5.07)	0–41.92
Shooting incidents rate	4.93 (4.81)	0–38.11
*Independent variables*		
Guns lost or stolen rate	0.39 (0.33)	0.04–2.03
Gun store rate	6.37 (11.21)	0–89.47
*(Sub-Sample of Jurisdictions with Gun Stores)*		
FFL1 per 100 k	8.61 (10.44)	0–79.65
FFL 2 per 100 k	1.38 (3.37)	0–28.58
Big box stores per 100 k	1.00 (1.28)	0–10.64
*Controls*		
Concentrated disadvantage	0 (1)	− 2.80–2.71
Gun homicide rate	1.08 (0.91)	0–3.71
Percent Black	8.87 (11.83)	0–76.54
Percent Hispanic	33.28 (22.89)	3.02–98.28
State	0.23 (–)	0–1

Table 2 provides the zero-order correlations. Focusing on the relationships between the independent variables and dependent variables, both rates of guns lost or stolen and gun stores were significantly positively associated with the number of citizens shot per capita as well as shooting incidents per capita—justifying a more thorough and rigorous examination in the multivariate analyses. At the bivariate level, jurisdictions with higher rates of guns reported lost or stolen and those with higher rates of gun stores tend to also have higher rates of citizens shot by police and shooting incidents; the association between the rates of gun stores and the outcomes is stronger than that of the rates of guns reported lost or stolen. All of the control variables, with the exception of the percentage of Hispanic residents and shooting incident rates, were also significantly positively associated with the outcomes of interest. Rates of both citizens shot by police and shooting incidents tend to be higher in jurisdictions with higher levels of concentrated disadvantage, gun homicide rates, percentages of Black and Hispanic residents, and those in the state of Florida.

**Table 2:** Zero-order correlations

	*Y*_1_	*Y*_2_	*X*_1_	*X*_2_	*X*_3_	*X*_4_	*X*_5_	*X*_6_	*X*_7_
*Y*_1_ citizens shot rate	–								
*Y*_2_ shooting incidents rate	0.99**	–							
*X*_1_ guns lost or stolen per 1000	0.33**	0.30**	–						
*X*_2_ gun stores per 100 k	0.49**	0.35**	0.24**	–					
*X*_3_ concentrated disadvantage^a^	0.45**	0.53**	0.33**	0.18**	–				
*X*_4_ gun homicide rate	0.32**	0.41**	0.27**	0.17**	0.51**	–			
*X*_5_ percent Black	0.31**	0.26**	0.28**	0.21**	0.42**	0.45**	–		
*X*_6_ percent Hispanic	0.07	0.19**	− 0.05	− 0.25**	0.58**	0.23**	− 0.16*	–	
*X*_7_ state (1 = Florida)	0.32**	0.23**	0.25**	0.46**	0.20**	0.12	0.55**	− 0.27**	–

*p < 0.05; **p < 0.01 (two-tailed test)

a = Weighted factor score of 3 items: percentage of the population living in poverty, percentage of the population with female-headed households, and the median income (reverse coded)—measured for each city

A few other bivariate relationships between the covariates are worth noting. Both the rates of guns reported lost or stolen (*r* = 0.27; *p* < 0.01) and gun stores (*r* = 0.17; *p* < 0.01) are significantly positively associated with the logged gun homicide rate in cities, although the former’s correlation is stronger. This mirrors prior work (Semenza et al. 2021) that has found illicit firearm availability is more closely associated with general homicide rates compared to specific types of homicide (e.g., intimate partner) where legal firearm availability is more salient. There is also a positive relationship between the rates of guns stores and guns reported lost or stolen at the city level (*r* = 0.24; *p* < 0.01).

Table 3 presents the negative binomial regression results, which include both incidence rate ratios (IRR) and confidence intervals (CI). Models 1–3 examine the first set of dependent variables—pooled counts of citizens shot by police—while models 4–6 examine the pooled counts of police shooting incidents. Each set of models explore the independent variables separately before including both together (e.g., models 3 and 6). Models 1 and 4 show that the association between rates of guns reported lost or stolen and the police shooting outcomes do not persist at the 0.05 alpha level net of controls. On the other hand, and turning to models 2, 3, 5, and 6, jurisdictions’ rates of guns stores remain significantly associated with both the total number of citizens shot by police and police shooting incidents with the inclusion of the covariates—although the IRRs for these FFL rates are small (1.02). Levels of concentrated disadvantaged and, to a lesser extent, gun homicide rates are also more consistently positively and substantively associated with the number of citizens shot by police and shooting incidents in the multivariate analyses.

**Table 3:** Negative binomial regression analyses of police shooting counts (full sample)

Variable	Model 1		Model 2		Model 3		Model 4		Model 5		Model 6	
	Citizens shot		Citizens shot		Citizens shot		Shooting incidents		Shooting incidents		Shooting incidents	
	IRR	CI	IRR	CI	IRR	CI	IRR	CI	IRR	CI	IRR	CI
Guns lost/stolen rate	1.30^+^	0.98–1.72	–		1.29^+^	0.99–1.68	1.25	0.94–1.65	–		1.23	0.95–1.60
Gun stores rate	–		1.02***	1.01–1.03	1.02***	1.01–1.02	–		1.02***	1.01–1.03	1.02***	1.01–1.02
Concentrated disad.^a^	1.60***	1.36–1.88	1.51***	1.29–1.76	1.44***	1.22–1.69	1.62***	1.38–1.91	1.52***	1.30–1.78	1.46***	1.24–1.72
Gun homicide rate	1.16*	1.01–1.32	1.15*	1.01–1.31	1.13^+^	0.99–1.28	1.16*	1.01–1.32	1.14*	1.00–1.29	1.12^+^	0.99–1.28
Percent Black	0.99*	0.98–0.99	1.00	0.98–1.01	0.99	0.98–1.01	0.99*	0.98–1.00	1.00	0.99–1.00	1.00	0.99–1.01
Percent Hispanic	1.00^+^	0.99–1.00	1.00	0.99–1.00	1.00	0.99–1.01	0.99^+^	0.99–1.00	1.00	0.99–1.00	1.00	0.99–1.01
State	1.57**	1.21–2.04	1.24	0.95–1.62	1.27^+^	0.97–1.66	1.47**	1.13–1.91	1.15	0.88–1.50	1.18	0.90–1.55
LR Chi^2^	107.28***		122.99***		121.94***		103.06***		120.48***		118.06***	
Pseudo R-squared	0.09		0.10		0.11		0.09		0.10		0.10	
N	233		242		233		233		242		233	

Entries include incidence rate ratios (IRR) and confidence intervals (CI)

^+^*p* < 0.10; *p < 0.05; **p < 0.01; ***p < 0.001 (two-tailed test)

a = Weighted factor score of 3 items: percentage of the population living in poverty, percentage of the population with female-headed households, and the median income (reverse coded)—measured for each city

Next, we explored the sub-sample of jurisdictions that had federally licensed gun stores. Table 4 provides the results from those analysis with separate models for rates of FFL1 stores, FFL2 stores, and Big Box stores. Similar to the full sample, models 1–3 examine the pooled counts of citizens shot by police, whereas models 4–6 examine the pooled counts of shooting incidents. Across all six models, rates of the three different types of gun stores per capita (e.g., FFL1, FFL2, and Big Box Stores) continue to be significantly positively associated with both citizens shot by police and police shooting incidents—again with relatively weak IRRs: 1.02 for FFL 1s, 1.07 for FFL2s, and 1.14–1.15 for Big Box Stores. When the most stringent alpha level of 0.001 is considered, rates of FFL2s (e.g., pawnshops) in models 2 and 5 are the most consistent statistically significantly associated types of gun stores. Rates of guns reported lost or stolen emerge as either significantly positively associated (*p* < 0.05; model 2) or marginally significant (*p* < 0.10; model 5) with the outcomes in two of the six models, but they certainly drop out when relying on more stringent alpha levels of 0.01 or 0.001. Levels of concentrated disadvantage and the gun homicide rate also remain the more consistent and substantive associations with the dependent variables.

**Table 4:** Negative binomial regression analyses of police shooting counts (sub-sample of jurisdictions with gun stores)

Variable	Model 1		Model 2		Model 3		Model 4		Model 5		Model 6	
	Citizens shot		Citizens shot		Citizens shot		Shooting incidents		Shooting incidents		Shooting incidents	
	IRR	CI	IRR	CI	IRR	CI	IRR	CI	IRR	CI	IRR	CI
Guns lost/stolen rate	1.20	0.90–1.61	1.32*	1.01–1.76	1.21	0.90–1.63	1.14	0.86–1.52	1.27^+^	0.96–1.67	1.14	0.85–1.54
FFL1 rate	1.02**	1.01–1.03	–		–		1.02***	1.01–1.03	–		–	
FFL 2 rate	–		1.07***	1.04–1.10	–		–		1.07***	1.04–1.10	–	
Big box store rate	–		–		1.14**	1.05–1.25	–		–		1.15**	1.05–1.25
Concentrated disad.^a^	1.45***	1.20–1.75	1.47***	1.23–1.76	1.45***	1.20–1.74	1.46***	1.21–1.77	1.49***	1.25–1.78	1.47***	1.22–1.76
Gun homicide rate	1.20*	1.02–1.42	1.25**	1.06–1.46	1.25**	1.06–1.47	1.20*	1.02–1.42	1.24**	1.06–1.46	1.25**	1.06–1.47
Percent Black	1.00	0.98–1.01	.99	0.98–1.00	0.99	0.98–1.01	1.00	0.98–1.01	0.99	0.98–1.00	0.99	0.98–1.01
Percent Hispanic	1.00	0.99–1.01	1.00	0.99–1.00	1.00	0.99–1.00	1.00	0.99–1.01	1.00	0.99–1.00	1.00	0.99–1.00
State	1.23	0.92–1.66	1.17	0.88–1.56	1.44*	1.09–1.91	1.14	0.82–1.53	1.08	0.81–1.45	1.34*	1.01–1.77
LR chi^2^	100.04***		107.39***		93.94***		96.94***		103.58***		91.34***	
Pseudo R-squared	0.11		0.12		0.11		0.11		0.12		0.10	
N	152		152		154		152		152		154	

Entries include incidence rate ratios (IRR) and confidence intervals (CI)

^+^*p* < 0.10; *p < 0.05; **p < 0.01; ***p < 0.001 (two-tailed test)

a = Weighted factor score of 3 items: percentage of the population living in poverty, percentage of the population with female-headed households, and the median income (reverse coded)—measured for each city

## Discussion

We set out to examine the relationship between two distinct measures of firearm availability (legal access via FFLs and illicit access via lost/stolen guns) and two measures of police shootings. Since all guns originate from FFLs, these two measures do not necessarily represent mutually exclusive pathways; however, we conceptualized FFLs as source of broad availability where people buy guns legally separately from the pool of guns that have been lost or stolen from an exploratory standpoint. The results affirm that legal firearm availability is a correlate of such shootings, alongside established correlates like concentrated disadvantage and existing gun crime. We found that the frequency of citizens shot by police, and shooting incidents are heightened in jurisdictions with a higher rate of licensed gun stores, including FFL1 and FFL2 dealers. On the other hand, we found no evidence to support the assertion that greater availability of illicit firearms is associated with an increase in police-involved shootings, which is both an interesting and counterintuitive finding since illicit firearms are the primary drivers of gun violence among the general population (Semenza et al. 2021; Braga et al. 2021; Cook et al. 2007; Papachristos et al. 2013). In fact, there is scant systematic information on the types of firearms possessed by citizens—legally obtained versus not—in shootings both by and of police officers. All firearms sold in the United States first make their way to the public through FFLs and our results suggest that this association between the rate of federally licensed gun stores at the city level is more consequential for police shootings than that of rates of guns lost or reported stolen.

These findings, however, merit closer scrutiny. The association between rates of gun stores and police shootings at the city level is relatively small and weak, which suggests there are other factors involved in police shootings that have less to do with levels of firearm availability or prevalence—at least according to our proxy measures. The results, instead, point to the fact that perhaps factors related to concentrated disadvantage are more relevant, such as persistent and exacerbated mental health challenges as well as higher levels of distrust of police and legal cynicism in those places. Due to data limitations, there were other unobserved and unmeasured factors of police departments and their officers that could not be accounted for in the statistical models (see footnote #4). They include variation in police training (e.g., de-escalation) as well as administrative policy governing officers’ firearms use (e.g., limiting shooting at moving vehicles). Future research should continue to focus on all of these avenues of inquiry.

There may be several reasons—albeit purely speculative—why gun stores are significantly associated with higher rates of police shootings. First, federally licensed stores may indicate higher legal gun ownership among citizens who interact with the police, including in their homes, in motor vehicles, and on the streets. All policing is territorial-based, and this study adds to a growing literature that suggests officers’ firearms use is likely influenced by the broader social context of their working conditions related to encountering firearm threats from citizens. Gun stores, related advertising, and the volume of people patronizing those stores may also reflect a broader cultural demand for guns that local police are trained to assess. The presence of more legal gun stores may feed secondary illegal markets in a city, via straw purchases or thefts; there was a significant positive correlation between a city’s rate of gun stores and respective rates of guns lost or reported stolen. The use of stolen guns in a crime may further escalate police-citizen interactions. Additionally, citizens in areas that experience higher gun crime may want to purchase firearms for their protection; thus, increasing the demand and rates of FFLs in those cities. While there are likely several mechanisms at play, the results here suggest that greater legal gun availability in a community contributes to higher police shooting incidents, above and beyond the number of guns reported lost and stolen, but the substantive impact of FFL rates does pale in comparison with other variables—namely concentrated disadvantage and the gun homicide rate—in the statistical models.

From a policy perspective, and apart from striving to address broader macrosocial characteristics like reducing economic disadvantage that are not only difficult but longer-term investments (MacDonald 2022), one of the more proximal changes could ensure that federally licensed dealers are appropriately monitored and audited to prevent dealers from engaging in illicit behavior, such as selling to straw purchasers or individuals prohibited from owning a firearm. Of course, resources are scarce and there is some concern that—given the small associations of FFLs with the outcomes—those resources could have a larger impact elsewhere. However, perhaps, a targeted approach to stricter surveillance and monitoring of higher-risk FFLs (e.g., those with previous violations; FFL2s) is more cost effective and cost efficient. Ultimately, this can help prevent firearms sold through dealers from making their way into secondary markets while also keeping legally accessible guns out of the hands of people that are most likely to engage in violent encounters. This may be especially salient for individuals who have prior violent or domestic offenses on their record. Only an estimated 12–40% of gun dealers are audited by ATF in a given five-year period and most dealers found to be negligent or non-compliant with federal law do not have their licenses revoked (Department of Justice 2022; Freskos et al. 2022). Consistent auditing and clear guidelines for license revocation can help support supply side efforts to reduce the presence of firearms in police encounters that may ultimately lead to police shootings.

Greater transparency and closer communication between local firearm dealers and law enforcement agencies can also help ensure dealers are selling firearms properly while providing police with proper insight into citizen firearm access more broadly. Our results show that access to firearm dealers is more closely associated with police shootings than the supply of illicit firearms, albeit a proxy measure, in a given jurisdiction. As such, it may be particularly important for police to be aware of firearms purchased at local dealers through licensing and permitting mechanisms currently required in twenty-five US states for concealed carry (Doucette et al. 2022; Donohue et al. 2019). For example, if police officers are aware of a firearm permit where a gun has been purchased by an individual during a traffic stop or residential visit, they may be able to better assess a threat and reduce the likelihood of a shooting during an altercation. Research shows that states without permitting requirements for carrying a firearm have higher rates of violent crime in general and this may well be similarly applicable to police shootings of citizens (Donohue et al. 2019).

Our study has several limitations which provide opportunities for future research. First, the sample of cities used in our analysis is not nationally representative, although California and Florida do represent two of the three largest states in the country, covering almost 60 million Americans. Second, we pooled estimates to conduct a cross-sectional—rather than longitudinal—analysis of the association between gun availability in these states and police shootings. This approach is common in research using a dependent variable with rare occurrence, but the decision limits our ability to make any causal inference between gun availability and police shootings. The inability to make causal inferences also applies to any other variables that were included, such as the relationship between higher rates of gun crime potentially influencing more gun stores in a city due to an increase in demand for firearms for defensive purposes. Third, and due to the aforementioned lack of data for nonfatal police shootings of citizens, we had to rely on different pooled time periods for Florida’s (2009–2014) and California’s (2016–2021) outcomes—not mention the lag between the independent variables and California’s dependent measures. We cannot definitively state that the measures of gun availability or police shootings of citizens are time-invariant, and must acknowledge the growing national concern over deadly police force and subsequent reform efforts starting in the summer/fall of 2014 in the wake of the deaths of Michael Brown in Ferguson, Missouri and Eric Garner in New York (continuing with the murder of George Floyd in summer 2020) that bisects the pooled outcome time periods between Florida and California. Lastly, in using a city level analysis to examine our research question, we recognize that there could be more localized impacts of gun stores at smaller units of analysis (Semenza et al. 2022b). For instance, the effects of attendant advertising, traffic flow, and people carrying cash when patronizing gun stores could alter crime and police behavior. Future spatial analyses could help further understand whether police shootings cluster nearby particular gun store locations.

## Conclusions

Despite these limitations, this study expanded on the previous literature in three ways: extending beyond fatal police shootings to also include nonfatal injurious incidents, employing smaller units of analysis at the city level, and exploring proxy measures for both legal and illicit firearm availability. It identified the presence of local firearm dealers as a significant correlate of police shootings even after accounting for the availability of illicit firearms in the area and other factors consistently associated with police firearms use. Reducing gun violence in all forms is a major task for public health in the US, and the reduction in police shootings must be part of the agenda. Although supply side efforts to ensure proper dealer selling practices and improve police knowledge of local firearms access may help reduce the risk of shootings during a police-citizen altercation, ongoing research is necessary to identify additional interventions to support the goal of limiting both fatal and injurious police shootings throughout the country. It is necessary to bring down police shootings in the US and one way to do so may be through a supply side focus that considers levers related to dealers and gun markets.

### Supplementary Information

**Additional file 1:** Supplemental Materials.

## Data Availability

All data and measures used for analyses were publicly available. More information on where to locate the data/measures and how variables were operationalized are included in the Supplemental Materials file.

## Abbreviations

FFL: Federal firearms licensees (i.e., federally licensed firearms dealers)
US: United States
PTP: Permit to purchase
ATF: Bureau of Alcohol, Tobacco, Firearms, and Explosives
ACS: American Community Survey
NCIC: National Crime Information Center

## Footnotes

1 Another study (Swedler et al. 2015) performed a similar state-level analysis examining rates of felonious homicides of police officers who were killed with firearms, which found that states with higher legal firearm ownership was associated with higher rates of felonious homicides of officers.

2 For example, one study (Shjarback 2019) found that 51% of citizens shot by police in Texas in 2016–2017 survived their gunshot wounds. Another study (Nix and Shjarback 2021) found 45% of those struck by police gunfire in four states did not die and a national analysis (Ward 2023) from 2015 to 2020 found only 55% of citizens struck by police gunfire died with, on average, 790 people nonfatally injured by police gunfire each year.

3 See the Supplemental Materials file for more information on the data sources, particularly The Trace’s “Missing Pieces”, measurement of key variables, and replication materials.

4 An effort was made to obtain police department measures as statistical controls, including firearm policies and documentation requirements, from the 2016 Law Enforcement Management and Administrative Statistics (LEMAS) survey. However, only 113 of the 253 respective agencies were included in the 2016 LEMAS survey (44.7%).

5 A separate set of multivariate models using the standardized rates of citizens shot by police and shooting incidents as dependent variables, which employed ordinary least squares (OLS) regression analysis, is presented in the Supplemental Materials file. These OLS findings are relatively consistent with the negative binomial models.
