# Supplementary material for: Firearm availability and police shootings of citizens: a city level analysis of fatal and injurious shootings in California and Florida
Source: Inj Epidemiol. 2023 Oct 20;10:50. doi: 10.1186/s40621-023-00466-1 (PMC10588120; doi:10.1186/s40621-023-00466-1)
Supplement: Supplementary file 1 — Additional file 1: Supplemental Materials. [file 40621_2023_466_MOESM1_ESM.docx]

**SUPPLEMENTAL MATERIALS**

Data Sources 2

Cities in the Sample 6

Supplemental Multivariate Analyses 7

Replication Materials Information 9

**Data Sources**

Dependent Variables

*Florida*

More information about and access to the publicly available *Tampa Bay Times*’ “Why Cops Shoot” database, including its sophisticated and diverse methodology, can be found at:

<https://projects.tampabay.com/projects/2017/investigations/florida-police-shootings/>

Additionally, the database possesses easy to search tools such as “city”, which can be found here:

<https://projects.tampabay.com/projects/2017/investigations/florida-police-shootings/database/>

All of the raw data can be found at:

<https://github.com/tbtimes/florida-police-shooting-data>

*California*

California’s URSUS police use of force reporting system was created by the state legislature, which passed Assembly Bill (AB) 71 and added Government Code (GC) section 12525.2 in late 2015. It “mandates law enforcement agencies (LEA) in California to report use of force incidents that result in serious bodily injury or death or involved the discharge of a firearm. Effective January 1, 2016, all LEAs were required to begin collecting data on use of force incidents for submittal to the Department of Justice (DOJ).” We focused only on police officers’ discharges of a firearm and excluded all “shoot and miss” cases; only those shooting incidents where a person was shot and injured or killed were included. More information as well as the publicly available URSUS data can be accessed at:

<https://openjustice.doj.ca.gov/data>

For each “civilian-officer” file from 2016-2021, we filtered out only those use of force cases where a police officer discharged his/her firearm and a citizen was struck by gunfire:

“Filter (Civilian); Filter (Discharge of Firearm_individual-True); Filter (Received_Force_Type-all where “Disccharge of firearm (hit) = checked)”

The “Incident_ID” variable for each of the “civilian-officer” files was then used to merge with the “incident” files to identify which particular city the police shooting incident took place in.

*Attempting to Validate the Dependent Variables*

We attempted to validate the both the Florida and California measures by comparing the fatal police shootings of citizens to other crowdsourced, media-based national data collections. Unfortunately, however, this was not possible to do with the non-fatal, injurious shootings since of these datasets only capture fatalities (e.g., The Washington Post’s Fatal Force, Fatal Encounters).

Florida: Since *The Washington Post*’s Fatal Force database begins documentation on January 1, 2015 and the *Tampa Bay Times*’ measures of police shootings run from 2009-2014, we needed to use the Fatal Encounters database in order to compare and match to. A case-by-case comparison between the *Tampa Bay Times* and Fatal Encounters for each fatal shooting was conducted – finding that the *Tampa Bay Times* was only missing 1 fatal shooting compared to Fatal Encounters (279 out of 280; 99.6%)

California: Given that *The Washington Post’*s Fatal Force database begins documentation on 1/1/2015 and California’s measures run from 2016-2021, a case-by-case comparison between the fatal police shootings of citizens in the URSUS and both *The Washington Post* and Fatal Encounters was conducted – finding that 591 out of 601 (98.3%) fatal shootings could be validated using the other two data sources.

Taken as a whole, 870 out of the 881 fatal police shootings of citizens in the dataset (98.8%) could be validated using the two other data sources that also catalogue fatal police shootings.

*Creating Rates of 1) Citizens Shot by Police and 2 Police Shooting Incidents*

Because the Florida data cover years 2009-2014 and the California data cover years 2016-2021, we wanted to standardize rates of both citizens shot by police and police shooting incidents using the most accurate time-appropriate estimates of each jurisdiction’s population.

Using the U.S. Census Bureau’s Advanced Search tool, we employed the American Community Survey’s 5-year estimates for total population. More specifically, Tables DP05 (ASC Demographic and Housing Estimates) were used: 2011 (5-year estimates of 2009-2013) for Florida’s jurisdictions and 2018 (5-year estimates of 2016-2020) for California’s jurisdictions.

These rates were used for descriptive and zero-order correlation purposes. In addition, they were used as the dependent variables for the supplemental analyses

Independent Variables

*The Trace’s “Missing Pieces”*

The measure of illicit firearm availability in cities comes from The Trace’s “Missing Pieces” database. It combines over 800,000 records on guns reported lost, stolen, or recovered by law enforcement agencies in major cities and counties in 36 states. The Trace is an independent nonprofit news organization that compiled the data through public records requests. These requests were filed with the assistance of more than a dozen NBC TV stations along with the editors at The Trace, with a focus on the property and evidence-management systems of major cities around the US. The data can be accessed at:

<https://www.thetrace.org/missing-pieces-data/>

The “Missing Pieces” data, as a whole, have not been independent validated in other peer-reviewed articles. However, the measures do reflect broader national trends in guns reported lost and stolen as shown by the National Crime Information Center (NCIC):

<https://www.thetrace.org/2017/11/stolen-guns-violent-crime-america/>

Because guns recovered by police may be more indicative of an agency’s level of proactivity (as opposed to illicit firearm availability in the community), we elected to remove those numbers from the measures; only guns lost and reported stolen to police were included in our variables.

The Trace’s “Missing Pieces” collected such measures from 2010 through 2017. Years 2010-2016 are consistently reported; however, 2017 had issues with missing data. For example, in 2017, 45 fewer cities in California reported measures along with 24 fewer cities in Florida. In an effort to keep the measurement of this independent variable consistent, we elected to create the same five-year average (2010-2014) rates for cities in both states rather stagger California’s cities by a few years (e.g., 2012-2016).

*Bureau of Alcohol, Tobacco, Firearms, and Explosives (ATF)*

The measure of legal firearm availability comes from the number of federally licensed firearm dealers (FFLs) in a city – compiled by ATF. ATF’s FFL Listing provides a complete list of FFLs and their locations in the US on their website, which can be accessed at:

<https://www.atf.gov/firearms/listing-federal-firearms-licensees>

The website allows for FFL Listings to be searched from 2014 through present day; however, ATF once listed data from 2010-2013 and were obtained by the authors previously. Years 2010-2013 have since been removed from the website, but those measures are available upon request.

**Cities in the Sample**

*California* (n = 196): Alameda; Alhambra; Anaheim; Antioch; Arcadia; Atascadero; Atwater; Azusa; Bakersfield; Baldwin Park; Banning; Barstow; Beaumont; Bell Gardens; Belmont; Benicia; Berkeley; Beverly Hills; Blythe; Brawley; Brea; Buena Park; Burbank; Burlingame; Calexico; Carlsbad; Ceres; Chico; Chino; Chula Vista; Citrus Heights; Claremont; Clovis; Colton; Compton; Concord; Corcoran; Corona; Costa Mesa; Covina; Culver City; Cypress; Daly City; Davis; Delano; Desert Hot Springs; Dinuba; Downey; Dublin; East Palo Alto; El Cajon; El Centro; El Cerrito; Elk Grove; Escondido; Eureka; Fairfield; Folsom; Fontana; Foster City; Fountain Valley; Fremont; Fresno; Fullerton; Gardena; Gilroy; Glendale; Glendora; Hawthorne; Hayward; Hemet; Hercules; Hollister; Huntington Beach; Huntington Park; Indio; Inglewood; Irvine; Laguna Beach; Lemoore; Lincoln; Livermore; Lodi; Lompoc; Long Beach; Los Altos; Los Angeles; Los Banos; Los Gatos; Madera; Manhattan Beach; Manteca; Martinez; Maywood; Menlo Park; Merced; Millbrae; Milpitas; Modesto; Monrovia; Montclair; Montebello; Monterey; Monterey Park; Morgan Hill; Murrieta; Napa; Newark; Newport Beach; Novato; Oakdale; Oakland; Oceanside; Ontario; Orange; Oxnard; Pacifica; Palm Springs; Palo Alto; Paradise; Paramount; Pasadena; Patterson; Petaluma; Pittsburg; Placentia; Pleasant Hill; Pleasanton; Pomona; Porterville; Port Hueneme; Redding; Redlands; Redondo Beach; Redwood City; Reedley; Rialto; Richmond; Ridgecrest; Riverbank; Riverside; Rocklin; Rohnert Park; Roseville; Sacramento; Salinas; San Bernardino; San Bruno; San Carlos; San Diego; San Fernando; San Francisco; San Gabriel; San Jacinto; San Jose; San Leandro; San Luis Obispo; San Mateo; San Pablo; San Rafael; San Ramon; Santa Ana; Santa Barbara; Santa Clara; Santa Cruz; Santa Maria; Santa Paula; Santa Rosa; Seaside; Selma; Simi Valley; Soledad; South Gate; South Lake Tahoe; South Pasadena; South San Francisco; Stockton; Suisun City; Torrance; Tracy; Tulare; Turlock; Tustin; Union City; Upland; Vacaville; Vallejo; Visalia; Walnut Creek; Watsonville; West Covina; Westminster; Whittier; Windsor; Woodland; Yuba City

*Florida* (n = 57): Apopka; Aventura; Boca Raton; Boynton Beach; Bradenton; Cape Coral; Clearwater; Coconut Creek; Coral Gables; Coral Springs; Delray Beach; Doral; Fort Lauderdale; Fort Myers; Gainesville; Hallandale Beach; Hialeah; Hollywood; Homestead; Jacksonville; Jacksonville Beach; Key West; Kissimmee; Lakeland; Largo; Lauderhill; Margate; Melbourne; Miami; Miami Beach; Miami Gardens; Miramar; North Miami; North Miami Beach; North Port; Ocala; Orlando; Palm Bay; Palm Beach Gardens; Panama City; Pembroke Pines; Pensacola; Pinellas Park; Plantation; Port St. Lucie; Riviera Beach; Rockledge; St. Petersburg; Sarasota; Sunrise; Tallahassee; Tampa; Titusville; West Palm Beach; Winter Garden; Winter Park; Winter Springs

**Supplemental Multivariate Analyses**

Aside from the series of negative binomial regression models presented in the paper, we also ran a series of supplemental multivariate analyses using both the standardized rates of citizens shot by police and shooting incidents. Given that both sets of outcomes were measured continuously the supplemental analyses utilized ordinary least squares (OLS) regression.

In their current form, the two dependent variables were positively skewed. As a result, they were both log transformed after adding a constant of 1. This reduced the skew (i.e., skewness divided by the standard error of skewness) from 16.83 to -2.25 for the citizens shot rate and from 14.98 to -2.25 for the shooting incidents rate.

A number of model diagnostics were performed to best ensure that the parameter estimates were unbiased. Although many of the controls were highly correlated with one another – particularly concentrated disadvantage, gun homicide rates, and the racial/ethnic demographics – none exceeded the traditional 0.70 threshold. Additionally, variance inflation factors for each model were all below 3. Breusch-Pagan tests for heteroskedasticity revealed that some models did not hold constant variances. In those cases, the models were run with Huber-White robust standard errors to account.

**Replication Materials Information**

All replication materials have been posted on the first author’s personal website. They can be found at:

https://www.johnshjarback.com/replication-materials

They include both SPSS and STATA versions of the data as well as a syntax file (used in SPSS for creating and recoding variables) and a Do-file of all code for STATA.

Table S1 – OLS Regression Analyses of Police Shooting Rates (Full Sample)

| Variable | Model 1  Citizens Shot Rate  *b* (SE) [β] | Model 2  Citizens Shot Rate  *b* (SE) [β] | Model 3  Citizens Shot Rate  *b* (SE) [β] | Model 4  Shooting Incident Rate  *b* (SE) [β] | Model 5  Shooting Incident Rate  *b* (SE) [β] | Model 6  Shooting  Incident Rate  *b* (SE) [β] |
| --- | --- | --- | --- | --- | --- | --- |
| Guns Lost or Stolen Rate | .21 (.14) [.09] | -- | .21 (.14) [.08] | .17 (.14) [.07] | -- | .16(.13)[.07] |
| Gun Stores Rate | -- | .01^*^ (.01) [.19] | .01^*^ (.01) [.17] | -- | .01^*^ (.01) [.19] | .01^*^(.01)[.18] |
| Concentrated Disadvantage^a^ | .42^***^ (.07) [.50] | .40^***^(.07)[.47] | .36^***^(.07)[.43] | .44^***^(.07)[.53] | .41^***^(.07)[.49] | .37^***^(.07)[.45] |
| Gun Homicide Rate | .19^**^ (.07) [.21] | .18^**^(.07) [.19] | .17^*^ (.07) [.18] | .19^**^ (.07) [.21] | .17^*^ (.07) [.19] | .17^*^(.07)[.18] |
| Percent Black | -.01^**^ (.00)[-.21] | -.01 (.01)[-.12] | -.00 (.01)[-.14] | -.01^**^(.00)[-.20] | -.01(.01) [-.11] | -.01(.01)[-.13] |
| Percent Hispanic | -.00 (.00) [-.11] | -.00 (.00)[-.05] | -.00 (.00)[-.03] | -.00 (.00) [-.13] | -.00(.00) [-.07] | -.00(.00)[-.05] |
| State | .38^**^ (.13) [.20] | .15 (.15) [.08] | .24 (.15) [.12] | .32^*^ (.13) [.17] | .10 (.15) [.05] | .18 (.14) [.09] |
| F-test  R-squared  N | 26.94^***^  .36  233 | 28.53^***^  .38  242 | 24.88^***^  .38  233 | 26.38^***^  .35  233 | 28.91^***^  .38  242 | 24.59^***^  .37  233 |

Note: Entries include unstandardized coefficients (*b*) and standardized coefficients (β) in brackets with standard errors (SE) in parentheses.

*p < .05; **p < .01; ***p < .001 (two-tailed test).

a = Weighted factor score of 3 items: percentage of the population living in poverty, percentage of the population with female-headed households, and the median income (reverse coded) – measured for each city.

Table S2 – OLS Regression Analyses of Police Shooting Rates (Sub-Sample of Jurisdictions with Gun Stores)

| Variable | Model 1  Citizens Shot Rate  *b* (SE) [β] | Model 2  Citizens Shot Rate  *b* (SE) [β] | Model 3  Citizens Shot Rate  *b* (SE) [β] | Model 4  Shooting Incident Rate  *b* (SE) [β] | Model 5  Shooting Incident Rate  *b* (SE) [β] | Model 6  Shooting  Incident Rate  *b* (SE) [β] |
| --- | --- | --- | --- | --- | --- | --- |
| Guns Lost or Stolen Rate | .24^*^ (.12) [.12] | .32^*^ (.14) [.16] | .24 (.15) [.11] | .19^+^ (.11) [.09] | .27^+^ (.14) [.13] | .18 (.14) [.09] |
| FFL1 Rate | .02^*^ (.01) [.22] | -- | -- | .02^*^ (.01) [.24] | -- | -- |
| FFL 2 Rate | -- | .06^**^(.02) [.25] | -- | -- | .06^**^(.02) [.26] | -- |
| Big Box Store Rate | -- | -- | .09^*^ (.04) [.16] | -- | -- | .10^*^ (.04) [.17] |
| Concentrated Disadvantage^a^ | .33^***^(.08)[.39] | .34^***^(.08)[.41] | .36^***^(.08)[.44] | .33^***^ (.08) [.41] | .35^***^(.08)[.42] | .37^***^ (.08) [.45] |
| Gun Homicide Rate | .20^*^ (.08) [.22] | .21^**^(.07) [.23] | .21^**^(.07) [.24] | .20^*^ (.08) [.22] | .20^**^(.07) [.23] | .21^**^ (.07) [.24] |
| Percent Black | -.00 (.01) [-.08] | -.01^+^(.01)[-.13] | -.01(.01) [-.12] | -.00 (.01) [-.06] | -.01(.01) [-.12] | -.01 (.01) [-.10] |
| Percent Hispanic | -.00 (.00) [-.03] | -.00 (.00)[-.07] | -.01(.00) [-.07] | -.00 (.00) [-.05] | -.00(.00) [-.09] | -.00 (.00) [-.09] |
| State | .08 (.14) [.05] | .01 (.14) [.01] | .18 (.13) [.11] | .03 (.13) [.02] | -.04(.14) [-.02] | .13 (.13) [.08] |
| F-test  R-squared  N | 18.60^***^  .46  152 | 20.11^***^  .47  152 | 18.23^***^  .45  154 | 18.09^***^  .46  152 | 20.37^***^  .47  152 | 16.60^***^  .44  154 |

Note: Entries include unstandardized coefficients (*b*) and standardized coefficients (β) in brackets with standard errors (SE) in parentheses.

+p < .10; *p < .05; **p < .01; ***p < .001 (two-tailed test).

a = Weighted factor score of 3 items: percentage of the population living in poverty, percentage of the population with female-headed households, and the median income (reverse coded) – measured for each city.
